# Testing the efficacy of group cognitive-behavioral therapy for pathological internet use among undergraduates in Nigeria

**DOI:** 10.47626/2237-6089-2021-0348

**Published:** 2023-01-04

**Authors:** Moses Onyemaechi Ede, Chinedu Ifedi Okeke, Janet N. Igbo, Eucharia Aye

**Affiliations:** 1 Department of Education Foundations Faculty of Education University of the Free State Bloemfontein South Africa Department of Education Foundations, Faculty of Education, University of the Free State, Bloemfontein, South Africa.; 2 Department of Educational Foundations Faculty of Education University of Nigeria Nsukka Enugu Nigeria Department of Educational Foundations, Faculty of Education, University of Nigeria, Nsukka, Enugu State, Nigeria.

**Keywords:** Group cognitive-behavioral therapy, pathological internet use, college students, Nigeria

## Abstract

**Introduction:**

The aim of this study was to examine the effect of group cognitive-behavioral therapy (GCBT) on pathological internet use (PIU).

**Method:**

The study applied a group randomized controlled trial design to assign participants to intervention and control groups. A total of 40 college students aged 18 to 30 who were pathological internet users (PIUs) participated in this study and were randomly assigned to treatment and control groups. Participants completed a self-report scale entitled the Problematic Internet Use Scale (PIUS) at three time points. The intervention lasted 8 weeks. The data collected were statistically analyzed using repeated-measures analysis of variance (ANOVA).

**Results:**

The results showed that GCBT has significant efficacy, decreasing the symptoms of PIU among the GCBT participants compared to those in the control group and that the improvements were maintained at follow-up. We also found a significant interaction effect by time for PIU.

**Conclusion:**

From the study findings, we can conclude that GCBT has significant benefit for mitigating the severity of PIU in college students. Therefore, mental health professionals are encouraged to explore the benefits of GCBT in treating symptoms associated with PIU in school settings and beyond.

## Introduction

Pathological internet use (PIU) describes spending excessive resources (e.g., time, money, energy) on various activities on the internet to an extent that it might have harmful effects on the person’s physical and psychological condition, social, academic, professional, and interpersonal relationships, and other areas of life.^1 - 3^ Concepts such as excessive internet use, problematic internet use, internet addiction, internet abuse, unregulated internet use, and compulsive internet use have been used interchangeably with PIU.^4 , 5^

Like other addictions, PIU may be seen as an impulse-control disorder and can be linked to a variety of problems. PIU has characteristics like pervasiveness, depression, withdrawal, tolerance, and negative effects on daily functioning.^6 , 7^ Some health problems like headache and sleep pattern disruption might occur due to prolonged use of the internet.^8 , 9^ The sleep patterns of people with internet addiction are interrupted due to late night internet surfing leading to excessive fatigue, and impairment of the immune system, which leaves people with internet addiction prone to disease.^3 , 10^ Excessive use of the internet has led many students into risk of loss of significant relationships, sleeplessness, depression, and academic and social problems.^11 - 13^

Students’ overuse of the internet may interfere with their adaptive functioning, leading to depression as well as psychological disorders.^14 - 17^ Students have been considered vulnerable to relational problems because of over-accessibility of the internet.^18 , 19^ PIU among younger people has also been found to have a significant relationship with loneliness,^20 , 21^ negative self-esteem,^17^ symptoms of antisocial tendencies and externalizing control,^22^ shyness,^23^ and social disinhibition.^24^ As a result, it is important to help professionals to recognize the signs and symptoms of PIU and learn about some of the emerging treatment strategies for assisting college students with PIU disorder.^25^

Concern has been raised over excessive use of internet activities among college students in Nigeria.^26 - 28^ High levels of prolonged internet use and associated psychological and physical health problems including sleep disturbances and backache have been identified among students in colleges in Nigeria.^29 , 30^ Poor academic performance has also been linked to PIU among (Nigerian) students.^26 , 31^ Vices driven by PIU, including internet fraud and related crimes and vices, have attracted wide criticism in Nigeria. However, we found no studies attempting to practically address students with PIU problems in Nigeria. Some researchers have suggested application of therapeutic interventions to decrease the severity of PIU.^18 , 19 , 32 , 33^ The current study aims to employ cognitive-behavioral therapy to address PIU problems in a Nigerian college sample.

## Cognitive-behavioral therapy (CBT)

CBT^34^ is anchored in the assumption that reaction to automatic thoughts may lead to abnormality. Beck proposed that how people think (cognition), feel (emotion), and act (behavior) all interact to influence them.^34^ Automatic thoughts can cause negative consequences which may affect our cognitive, behavioral, and emotional responses.^34^ Beck et al.^35^ believed that how an individual feels and behaves is not anchored in life events, but in the way an individual responds to life events in that if the beliefs and responses are dysfunctional, the thoughts, attitudes, feelings, and reactions are likely to experience negative consequences. Beck suggested that for an individual to be functional and adaptive, their perceptions should reflect reality accurately.^35^ The CBT approach uses cognitive restructuring to change core thoughts and improve problem-solving skills towards acquiring desired adaptive behaviors. The CBT approach has been widely found to be beneficial in alleviating impulse control disorder.^36 - 40^ Because PIU could be accompanied and characterized by dysfunctional thoughts,^41^ it is sensible to support the view that CBT could be an effective intervention for decreasing severity of internet usage.

Although there is strong emerging evidence in the literature that CBT offers benefit for treating pathological online activities, the beneficial effects of CBT are still uncertain across cultures.^37^ This is because of some methodological weaknesses and cultural differences which exist among populations with PIU.^38^ Thus, further studies are required to evaluate the effectiveness of group CBT for PIUs in different cultural settings. Low-resource settings, such as the sub-Saharan region, are poorly represented in CBT literature. This study will try to address this gap by examining how the group CBT approach benefits people with PIU problems.

Using group cognitive-behavioral therapy may lead to desired behavioral outcomes.^42 , 43^ The effectiveness of groups for changing students’ maladaptive behaviors has been demonstrated.^42 - 44^ A group is one of the social platforms on which therapeutic dynamics and group processes can be received and revealed.^45 , 46^ Having similar characteristics as well as tendencies and interests to socialize might influence people to come together as a group. In essence, possessing common characteristics drives people to form a group in which they can interact, learn, and flourish interpersonally and socially.^47^

Studies are lacking on the effectiveness of group psychotherapeutic interventions for Nigerian college students who have PIU problems. Prior literature suggests that therapy taking a cognitive-behavioral approach can be used for treatment of impulse-control disorders like PIU,^48^ but its promising effects have not been demonstrated in the Nigerian student population. Therefore, the main objective of this study is to evaluate the effectiveness of group CBT for PIUs in Nigeria. Our study will provide additional evidence regarding the effectiveness of CBT in a poorly explored setting. Specifically, this study examines the effect of group cognitive-behavioral therapy on PIU among students in Nigeria. We hypothesized that group cognitive-behavioral therapy will be significantly effective in reducing PIU among students. We also hypothesized that the significant effect of group cognitive-behavioral therapy in reducing PIU among students will be sustained at follow-up evaluation.

## Methods

### Ethical approval

An ethical clearance letter was obtained from the research ethics committee at the Department of Educational Foundations, University of Nigeria. This study was carried out in line with the research ethics on human participants as specified by the American Psychiatric Association^49^ and in the Declaration of Helsinki. The provost of the College was informed of the study and approval was granted. The trial was registered retrospectively on the University Hospital Medical Information Network Clinical Trials Registry (UMIN-CTR) (trial no.: UMIN000035345).

### Design of the study

This is a group randomized controlled trial design which involved pre-testing and post-testing of participants. Participants were randomly assigned to an experimental group (i.e., GCBT group) or a control condition (i.e., waiting-list group).^50^

### Participants

The researchers recruited a total of 40 participants out of 82 students screened from December 2017 to February 2018. The adequacy of the sample size was ascertained using GPower version 3.1.1. Participants completed written informed consent. We used the Diagnostic Statistical Manual of Mental Disorders (DSM-V) and International Classification of Diseases 11 (ICD-11) criteria on classification of disorders^49 , 51^ to screen participants for inclusion with the assistance of clinical psychologists. Specifically, the dimension of DSM-V focused on PIU. The researcher also considered specific eligibility and inclusion criteria such as 1) using a mobile phone to chat with friends while working on the road, 2) being excited when in a gaming center, 3) repeated unsuccessful attempts to control betting, 4) borrowing money to bet, 5) betting money on online gaming weekly, 6) literate in smartphone usage and willingness to participate in the study, and 7) being readily available for the study.

**Figure 1:**
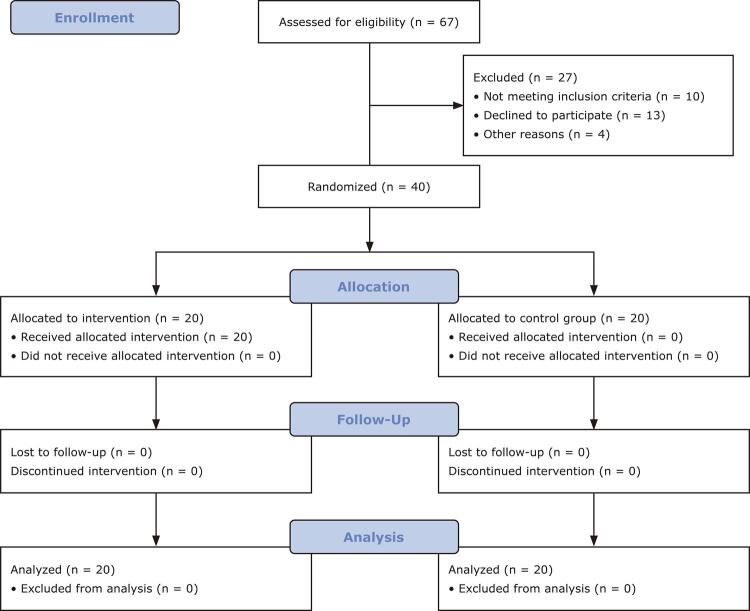
CONSORT flow diagram for allocation of participants

The revisions to these diagnostic health tools have clinically classified PIU as a mental and behavioral disorder.^52^ Exclusion criteria were ongoing psychological treatment, receiving medication, and not meeting the criteria for classification of the conditions in DSM-V and ICD-11. Specifically, the college students who were excluded due to psychological treatment and receiving medication were sick patients (e.g., with anxiety disorder). After the recruitment exercise, 40 participants were randomly assigned to two groups: the treatment group (n = 20) or a waitlist control group (n = 20) by the researchers. The randomization process adopted a simple random allocation sequence, using Random Allocation Software developed by Saghaei^53^ (see Figure 1 ) that was implemented by the researchers.

### Measure

The generalized Problematic Internet Use Scale 2 (GPIUS2) developed by Caplan^54^ is a 15-item self-report questionnaire that assesses generalized problematic internet use as it relates to cognitions, behaviors, and outcomes. The GPIUS2 is scored using a 7-point Likert type scale with response options ranging from 1 = strongly disagree to 7 = strongly agree. Lower scores represent less problematic level of internet use. The reliability and validity of the instrument have been ascertained in various cultural contexts. For example, Casale et al.^55^ utilized an Italian undergraduate population to report the internal consistency of the scale. Ferreira da Veiga et al.^56^ also reported good validity and reliability: α = 0.86. The current study established a Cronbach’s alpha co-efficient of α = 0.88.

### Procedures

The intervention was held after school hours and the treatment lasted for 8 weeks (i.e., 2 months), from 5th March, 2018 to 7th May, 2018, excluding a 1 month follow-up evaluation. The intervention was delivered at a Federal College of Education, Eha-Amufu in South-East Nigeria. Due to the college’s tight academic calendar, we were allowed to meet with the participants once a week. One session was held a week, lasting for 40 minutes. Different issues and topics were discussed during the sessions. In the first session, the researchers spelled out the procedure for group interaction. During this session we highlighted that our group norms included confidentiality, commitment, and mutual respect among participants.

The second session focused on describing the meaning of PIU. The participants, including the researchers, shared the meaning of PIU. This was an opportunity to understand the feelings of others and their experiences. The researchers also explored what needs participants had neglected as a result of excessive internet use. At the end of the session, a home exercise was given to the participants. At this stage, the researchers focused on dealing with basic factors that induce the participants to engage in excessive internet usage.

In session three, the participants were allowed to discuss the problems they encountered in their studies that could be linked with PIU. Some of these problems include inadequate preparation for examinations, late submission of assignments, inadequate concentration in classes, and examination phobia. The researchers exposed participants to cognitive behavioral techniques for possible solutions to the problems identified, such as problem solving, cognitive restructuring, and time management techniques. Other things the researchers trained the participants about were how to identify erroneous thoughts and cognitive errors, enhancing self-worth, controlling anger and assertiveness.^55^ Homework was given to participants to practice mood monitoring, and time management techniques.

During sessions four and five, the session participants shared knowledge on how to explore alternative activities rather than PIU and how to recognize internet usage patterns and their addiction triggers. This was followed by session six. The participants reviewed the group rules and made oral and written contracts with group members. They promised to make a commitment plan to quit excessive internet usage and committed to doing their assignments. In session seven, the researchers reviewed the practice exercises brought by the participants. Closely after that, they reviewed and made positive reminder cards and encouraged the group to use them in their real life, reducing excessive internet use. Assignment: use positive reminder cards.

Finally, the last session was held. The content was a follow up of the homework/assignment. Significant accomplishments of the group were reviewed. The group participants were thanked for their cooperation. Remind the group participants of the need for confidentiality. Refreshment was offered to the participants at the end the group sessions. The researchers expressed appreciation to the group participants for their commitment and cooperation. The techniques adopted during the treatment included shaping, cognitive restructuring, relaxation technique, systematic desensitization, reinforcement, ignoring technique, mood monitoring, problem-solving, and listening.

After completion of the experiment, both the treatment and the control group completed the post-test assessment. One month after completion of the post-test assessment, the 40 participants from both groups attended a one month follow-up meeting after which they completed the PIUs for the third time (Time 3) at the end of the meeting to ascertain if the probable effect was maintained and sustained by the participants. The maximum attendance was achieved because of active engagement by the research team, who monitored the intervention process. The participants saw them as external bodies monitoring their commitments, believing that they were under watch. Equally, the participants were provided with a hired bus that consistently conveyed them to the treatment venue. The presence of the college teachers/lecturers also enhanced the students’ active participation, as some of the lecturers were also committed to monitor the students’ activities. Given these precautions, no dropouts were recorded during the study. The researchers collated the data from the participants directly after each assessment. This is a blind study in which the researchers did not disclose the identities of the participants to the data analysts to avoid revealing which participants were in the intervention group and which were in the waitlisted group. This was to ensure concealment of information during the study. To ensure there were no missing responses, we engaged three data analysts, each to analyze one set of assessment data e.g., Time 1.

### Therapists

Two therapists were recruited and engaged for this study to deliver the intervention. They were two counseling psychologists trained in guidance and counseling who had both obtained Masters degrees. They were licensed to practice by the Counselling Association of Nigeria. They were within the age range of 32 to 45 years with about 5 years of experience. The researchers briefed them about the goal of the treatment and how to deliver the manual given to them.

### Treatment integrity

Based on the importance of effective and adequate implementation of the GCBT-program manual, we assigned two researchers who are also part of the research team to monitor the implementation processes of the intervention. Specifically, the integrity checkers or raters were designated to ensure that the therapists followed the guidelines and steps enshrined in the treatment manual. As part of their roles, they recorded the participants’ and therapists’ attendance. That is, the number of times each participant attended sessions. They took note of the number of times each participant asked and answered questions during the treatment sessions. They also monitored the participants’ and therapists’ behavioral and emotional responses.

### Treatment manual

The Group Cognitive Behavioural Therapy Manual (GCBT Manual) was developed by the researchers. The GCBT Manual covers an 8-week work plan that describes the steps the researchers used to administer treatment to the participants. It is organized as follows: sessions, objectives, content, treatment activities, and techniques. The sessions are sub-divided into eight sessions. The sessions were structured to last for 40 minutes each. The main objective is to reduce the pathological level of internet use among the students. The goal of each session is described in the manual, which contains the main topic(s) of each treatment session. Among the objectives of the treatment manual is to help participants appreciate the nature and problem of PIU, commit to genuinely engage in the GCBT program, understand the tenets of CBT theory and techniques, consider and adopt other activities to replace their intensive internet use, and identify antecedent behaviors and commit to developing specific plans to counter PIU. The activities of the researchers and participants, strategies, and materials are also included in the treatment manual (see Table 1 for summary). We developed the current manual taking cognizance of cultural differences and the fact that already existing manuals did not focus on PIU.

**Table 1:** Summary of group cognitive behavioral therapy (CBT) for pathological internet use (PIU)

Time frame (weeks)	Session	Topic	Activities	Techniques	Specific objective
1, 2	1, 2	Introduction, purpose, and rules; meaning of PIU.	Familiarization with the participants. Acquainted the participants with the purpose and relevance of the group process in improving students’ behaviors. Spelling out the boundary for group interaction. Making a contract on group norms such as confidentiality, commitment and treat each other with respect. Encouraged to think about, open up and discuss their concerns. Explained the meaning of PIU and basic needs for group formation. Explored their basic daily functioning that is impaired as a result of excessive internet use. Explored the factors of the internet addiction in terms of basic needs. Being conscious of symptoms of PIU and its management skills using CBT. Practice exercise was given to the participants.	Establishing therapeutic relationship; attending skills; listening skills; clarification; emotional disputation; cognitive restructuring; relaxation; reframing technique.	To help participants appreciate the nature and problem of PIU and commit to genuinely engage in the program. To understand and commit to genuine participation in group interaction.
3	3	Dealing with the consequences of PIU using CBT	Participants were allowed to discuss the problems they encountered in their study. Some of these problems include inadequate preparation for examinations, late submission of assignments, inadequate concentration in classes, and examination phobia. The researchers encouraged the participants to offer possible solutions to the problems identified. Explain briefly choice and CBT theories to the group members. Teach the group to use time management techniques. Homework assignment: apply time management techniques, practice exercise.	Cognitive disputation; time management; mood monitoring; restructuring; problem-solving skills; reflection of feeling; coping skills; thought monitoring and stopping; reframing technique.	Understand and admit that PIU has possibly, negatively impacted their lives as students. Understand the tenets of CBT theory and technique.
4	4	Exploration of alternative activities	Encourage the group to establish alternative activities. Present participants’ optional activities. Encourage the group members to use homework/assignment and self-help. Reviewed the timetables brought by individual participants. Guidelines for effective study habits were discussed. Homework/assignment.	Time management; assertive training; discussion; problem-solving skills; reframing technique.	Consider and adopt other activities to replace their intensive internet use.
5	5	Recognize internet usage pattern and their addiction triggers; help the group make a concrete plan to do better.	Review the group rules and follow up on the homework/assignment. Complete time plan form. Present it to the whole group. Reviewing previous discussion. Practice coping skills. Termination.	Discussion; proximity control; assertive training; interpretation.	Identifying antecedent behaviors and committing to developing specific plans to counter PIU.
6, 7	6, 7	Help the group make and use a verbal contract and positive reminder cards	Review the homework assignment. Make an oral or written contract with group members. Encourage the participants to make commitment plans. Make positive cues and encourage the group to use them in their real life. Homework/assignment: apply positive reminder cards. Reminding the participants of the last meeting.	Time management; proximity control; discussion; assertive training; interpretation; discussion, explanation.	Monitoring assimilation and encouraging the use of CBT techniques.
8	8	Revision of the program/termination	Follow up of the homework/assignment. Review significant accomplishments of the participants. Remind the group that even though the group experience has ended, confidentiality is still expected and important. Light and healthy refreshment is offered at the end the group session. Thank the group for their commitment and cooperation	Proximity control; discussion; interpretation; shaping and reinforcement.	Following up with participants on progress with techniques and any reported changes (positive or negative).

### Data analysis

The data received were subjected to analysis of variance (ANOVA). Partial eta squared was used to measure the effect size and to determine whether the two groups have similar standard deviations.^56^ The effect size was determined using limit number of *η_p_* ^2^ = 0.1 to 0.45 (small effect size), *η_p_* ^2^ = 0.46 to *η_p_* ^2^ = 0.67 (medium effect size), and *η_p_* ^2 ^= 0.68 and above (large effect size).^57^

## Results

Table 2 shows that the CBT group comprised 13 males (65%) and seven (35%) females; the waitlist control group comprised 17 males (85%) and three (15%) females. From the analyses of results, no significant gender difference was observed among the study participants (χ^2^ = 2.133, p = 0.144). In the CBT group, average mean age of participants was 23.20 ± 4.02 whereas in the waitlist control group, the average mean age of participants was 23.95 ± 3.83. No significant age difference was observed among the participants (t = 0.06, p = 0.55). However, the overall average was 23.57 ± 3.40 with skewness and kurtosis of 0.094 and -1.23 respectively. Regarding location, in the CBT group, seven participants (35%) were from rural areas, 13 (65%) were from urban areas. In the waitlist control group, five participants (25%) were from rural areas, 15 (75%) were from urban areas. No significant location difference was observed among the participants (χ^2^ = 0.476, p = 0.490). Concerning psychological problems, during the treatment, two (10%) of the participants had a withdrawal problem, three (15%) of the participants had an unsuccessful attempts to control problem, three (15%) of the participants had a loss of interest in academic activities problem, four (20%) of the participants had a loss of relationship problem, five (25%) of the participants had continued excessive use despite problems, three (15%) of the participants had a maladaptive coping problem. In the waitlist control group, two (10%) of the participants had a withdrawal problem, two (10%) of the participants had an unsuccessful attempts to control problem, three (15%) of the participants had a loss of interest in academic activities problem, three (15%) of the participants had a loss of relationship problem, six (30%) of the participants had continued excessive use despite problems, and four (20%) of the participants had a maladaptive coping problem. No significant difference was observed in psychological problems between the participants (χ^2^ = 0.577, p = 0.989). Concerning absence rate, in the treatment (CBT) group, 11 participants (55%) had a high rate and nine (45%) had a low rate; in the waitlist control group, eight participants (40%) had a high rate and 12 (60%) had a low rate. No significant difference was observed in absence rate between the participants (χ^2^ = 0.902, p = 0.342). Regarding average time spent online, in the treatment (CBT) group, six participants (30%) spent weekdays online and 14 (70%) spent weekends online; in the waitlist control group, seven participants (35%) spent weekdays online, and 13 (65%) spent weekends online. No significant difference was observed in average time spent online between the participants (χ^2^ = 0.114, p = 0.736). Concerning internet sport activities, in the treatment group, five (25%) of the participants were involved in Bet 9ja, two (10%) of the participants were involved in gaming, four (20%) of the participants were involved in Sure Bet, four (20%) of the participants were involved in Naira Bet, two (10%) of the participants were involved in Winner Golden, and three (15%) of the participants were involved in Merry Bet. In the waitlist control group, seven (35%) of the participants were involved in Bet 9ja, two (10%) of the participants were involved in gaming, three (15%) of the participants were involved in Sure Bet, three (15%) of the participants were involved in Naira Bet, three (15%) of the participants were involved in Winner Golden, and two (10%) of the participants were involved in Merry Bet. No significant difference was observed in internet sport activities between the participants (χ^2^ = 1.019, p = 0.961). Concerning level of study, in the treatment (CBT) group, six participants (30%) were in year 1, 10 (50%) were in year 2, and four (20%) were in year 3; in the waitlist control group, seven participants (35%) were in year 1, nine (45%) were in year 2, and four (20%) were in year 3. No significant difference was observed in level of study between the participants (χ^2^ = 0.130, p = 0.937). Regarding ethnicity, in the treatment (CBT) group, seven participants (35%) were of Igbo, three (15%) were of Hausa, five (25%) were of Yoruba, and five (25%) were of other ethnic backgrounds; in the waitlist control group, six participants (30%) were of Igbo, four (20%) were of Hausa, six (30%) were of Yoruba, and four (20%) were of other ethnic background. No significant ethnicity difference was observed among the study participants (χ^2^ = 0.422, p = 0.936).

**Table 2:** Sociodemographic and psychological characteristics of the participants

Characteristics	CBT group n (%)	Waitlist control group n (%)	Statistic	Sig
			**χ** ^**2**^	
Gender				
Male	13 (65.0)	17 (85.0)	2.133	0. 144
Female	7(35.0)	3 (15.0)		
Age *t* test				
Average age	23.20 ± 4.02	23.95 ± 3.83	0.604	0.550
Age range	18-30 years			
Skewness	0.094			
Kurtosis	-1.23			
Location χ^2^				
Rural	7 (35.0)	5 (25.0)	0.476	0.490
Urban	13 (65.0)	15 (75.0)		
Psychological problem				
Withdrawer	2 (10.0)	2 (10.0)	0.577	0.989
UC	3 (15.0)	2 (10.0)		
LIAA	3 (15.0)	3 (15.0)		
LR	4 (20.0)	3 (15.0)		
EUDP	5 (25.0)	6 (30.0)		
MC	3 (15.0)	4 (20.0)		
Absence rate				
High	11 (55.0)	8 (40.0)	0.902	0.342
Low	9 (45.0)	12 (60.0)		
Average time spent online				
Weekdays	6 (30.0)	7 (35.0)	0.114	0.736
Weekend	14 (70.0)	13 (65.0)		
Internet sport activities				
Bet 9ja	5 (25.0)	7 (35.0)	1.019	0.961
Gaming	2 (10.0)	2 (10.0)		
Sure Bet	4 (20.0)	3 (15.0)		
Naira Bet	4 (20.0)	3 (15.0)		
Winner Golden	2 (10.0)	3 (15.0)		
Merry Bet	3 (15.0)	2 (10.0)		
Level of study				
Year 1	6 (30.0)	7 (35.0)	0.130	0.937
Year 2	10 (50.0)	14 (45.0)		
Year 3	4 (20.0)	4 (20.0)		
Ethnicity				
Igbo	7 (35.0)	6 (30.0)	0.420	0.936
Hausa	3 (15.0)	4 (20.0)		
Yoruba	5 (25.0)	6 (30.0)		
Others	5 (25.0)	4 (20.0)		

% = percentage; CBT = Cognitive Behavioral Therapy; EUDP = continued excessive use despite problems; LIAA = loss of interest in academic activities; LR = loss of relationship; MC = maladaptive coping; n = number of participants; Sig = associated probability; *t* = independent sample *t* test; UC = unsuccessful attempts to control; χ^2^ = chi-square.

Table 3 reveals that the treatment and control groups did not vary significantly in their PIU results at Time 1, *F* (1,37) = 0.105, p = 0.902, *η_p_* ^2^ = 0.005, ∆R^2^ = 0.007 as measured by GPIUS2. According to the post-treatment and follow-up measures, group cognitive-behavioral therapy had a significant effect on the PIU scores of the students from the Federal Colleges of Education – *F* (1,37) = 887.321, p = 0.000, *η_p_* ^2^ = 0.908, ∆R^2^ = 0.914; and *F* (1,37) = 867.634, p = 0.000, *η_p_* ^2^ = 0.890, ∆R^2^ = 0.903. The results also showed that there was a significant interaction effect of time and treatment on the reduction of PIU scores among the students from the Federal Colleges of Education – *F* (2,33) = 30.43, p = 0.000, *η_p_* ^2^ = 0.451, ∆R^2^ = 0.463.

**Table 3:** Summary statistics for repeated-measures ANOVA showing the effect of GCBT on pathological internet use among undergraduate students by treatment condition and time

Outcome	Waitlist-control group (n = 20) Mean (SD)			Treatment group (n = 20) Mean (SD)			df	F	Sig	95%CI	*η_p_^2^*
	Time 1	Time 2	Time 3	Time 1	Time 2	Time 3					
PIUS	6.978 (2.95)	-	-	69.73 (2.12)	-	-	(1.37)	1.03	317	-0.145-0.436	0.013
PIUS	-	70.03 (2.78)	-	-	24.92 (1.60)	-	(1.37)	3953.37	0.000	-6.565--43.658	0.99
PIUS	-	-	70.34 (1.63)	-	-	24.13 (1.67)	(1.37)	7739.604	0.000	-7.268--45.140	0.99

*η_p_^2^* = partial eta squared (effect size); 95%CI = 95% confidence interval; df = degree of freedom; F = F-ratio; PIUS = Problematic Internet Use Scale; SD = standard deviation; Sig = significant value.

As shown in Table 3 , the values of *η_p_* ^2^(0.013, 0.99, and 0.99) at Time 1, Time 2, and Time 3 respectively showed that at baseline level there was no difference, while at post-test, participants in the treatment group experienced changes, and at follow-up, the change was maintained compared to those in waitlist-control group. This suggests that GCBT has a strong significant effect on reduction of PIU among undergraduate students. In order to ensure that the change found at Time 3 is reliable and to determine the strength of the change, a reliable change index (RCI) was conducted. The reliable change criterion result showed that there was clinically significant reliable improvement ( *RCCri t* = 1.96; standard error [SE] = 1.11). The result suggests that the clinically significant change in participants is reliable.

## Discussion

This study aimed at exploring the effect of GCBT on PIU among undergraduate students. Excessive use of the internet could cause academic, social, and interpersonal problems.^24^ After the intervention, the repeated-measures ANOVA statistic showed that GCBT helped to decrease PIU among college students. We also found that the effectiveness of GCBT was sustained at 1 month follow-up. The findings of this study also support Du et al.^58^ who indicated that group cognitive behavioral therapy decreased schooling adolescents’ internet addiction. Another study conducted in Germany revealed positive therapeutic effects of a cognitive behavioral group program in improving lifestyle of youngsters who were affected by PIU.^59^ Like the finding of the present study, an earlier study found a long-term effect of cognitive behavioral therapy on youth with PIU.^60^ The finding is also consistent with the study of Wartberg,^59^ who found that group therapy enhances significant change in people with PIU. This is in consonance with Kim^25^ who indicated that group therapy effectively reduced addiction level of internet addicted University students. In essence, PIUs that receive GCBT could change considerably. Previous evidence also indicated the efficacy of group therapy.^61 - 64^ Igwe^65^ also found that group psychotherapy is effective in reducing truancy record among students. This shows that the usefulness of group counseling cuts across cultures.

Given the efficacy of group psychotherapy, the present study supports that GCBT is useful in decreasing severity of internet pathology among undergraduate students. This is in line with Harry and Issack^63^ who explored the potential of online group counseling using Mauritian students with internet addiction disorder and found that 65% of the student population often thinks about what is happening on the internet. With regards to the vulnerability of internet addiction among students, mental health professionals are called to utilize GCBT to help students who have made unsuccessful efforts to control pathological internet usage.^63^ The finding of this study concurs with other Nigerian studies^62 , 64 - 66^ that found a significant role of GCBT in decreasing maladaptive behaviors and its efficacy for improved quality lifestyle. The finding of our study is also in line with that of Dowling et al.^38^ who suggested that CBT is a strong psychological treatment technique for PIU.

The results of the present study agree with past studies^67 - 71^ on the promising impacts of cognitive-behavioral therapy for decreasing psychological disturbances as well as improving quality of life over time. Like the long-term effect of rational-emotive and cognitive-behavioral therapy found by Ifeanyieze et al.,^72^ the treatment outcome of this study also suggested that CBT has long-term effectiveness in reducing PIU. Demonstrating the importance of psychological intervention of this kind, our study finding supports previous reports.^73 - 75^ that CBT helps people to reduce distorted thoughts irrespective of gender.

### Practice implications

Implications of this study are that GCBT could help in reducing PIU. Mental health professionals in practice could use GCBT in counseling students who use the internet excessively. Granted that the severity level of psychological symptoms associated with PIU in male and female college students could be minimized using GCBT, we encourage Nigerian public health professionals and other helping professionals to explore cognitive behavioral technique in reducing substance abuse. Since the study outcome revealed the long-term effects of CBT, cognitive behavioral therapists, psychologists, school counselors, teachers in colleges, and other schools should adopt the principles of CBT in helping students with behavioral problems.^76 - 79^ By practicing the assumptions of CBT, inappropriate behaviors such as pathological behaviors and the types in which students excessively indulge in could be reduced using CBT.

### Limitations of the study

One of the main limitations of the study is the use of the PIUS. Since the PIUS is not a mechanism of change tool, its use may affect the generalizability of the findings. Moreover, the scales are only quantitative measures. Participants were not well characterized in terms of cognitive performance, psychological problems, etc. We did not take into account other factors that could affect the results obtained. Future research could explore triangulation methods involving the assessment of significant others in monitoring change in PIU problems. It could be that certain individual/group dynamics were responsible for significant change in behavior rather than the GCBT. Our design was however not structured to identify the influence of different sessions. Future studies could isolate and monitor group and individual factors that may possibly influence treatment outcome. We also acknowledged that one of the major limitations of this study is its small sample and use of only Federal College of Education students. This methodological weakness tends to affect the generalizability of the results. We strongly advise readers of this journal to exercise caution in interpreting the outcome of this study. To that end, we encourage future studies to use larger samples. Given the limitations of this study, future studies that report the findings of the study should exercise caution in generalizing the study outcome to other groups in the education sector like teachers and other populations outside Nigeria.

## Conclusion

Owing to the prevalence of PIU, this study has investigated the effectiveness of GCBT on undergraduate college students with PIU. We found that GCBT significantly reduced PIU among the undergraduate college students. The result indicated that the effectiveness of GCBT on the college students’ PIU was sustained at follow-up assessment.
